# Correction: 2-Day versus C-reactive protein guided antibiotherapy with levofloxacin in acute COPD exacerbation: A randomized controlled trial

**DOI:** 10.1371/journal.pone.0253252

**Published:** 2021-06-09

**Authors:** The PLOS ONE Staff

## Notice of republication

An incorrect version of Fig 1 was published in error. The publisher apologizes for the error. This article was republished on June 1, 2021, to correct for this error. Please download this article again to view the correct version.

## References

[1] Mohamed Amine M, Selma M, Adel S, Khaoula Bha, Mohamed Hassene K, Imen T, et al. (2021) 2-Day versus C-reactive protein guided antibiotherapy with levofloxacin in acute COPD exacerbation: A randomized controlled trial. PLoS ONE 16(5): e0251716. 10.1371/journal.pone.0251716 34015041 PMC8136675

